# Effects of *Prunella vulgaris *on the Mice Immune Function

**DOI:** 10.1371/journal.pone.0077355

**Published:** 2013-10-30

**Authors:** Rui Huang, Min Zhao, Xingfen Yang, Junming Huang, Ying Yang, Bifeng Chen, Jianbin Tan, Jiankang Huang, Zhi Li, Yingjian Lv, Guiyuan Ji

**Affiliations:** 1 Guangdong Provincial Centre for Disease Control and Prevention, Guangzhou, People’s Republic of China; 2 Guangdong Provincial Institute of Public Health, Guangzhou, People’s Republic of China; 3 School of Public Health, Sun Yat-Sen University, Guangzhou, People’s Republic of China; Kaohsiung Chang Gung Memorial Hospital, Taiwan

## Abstract

The present study was designed to evaluate the effects of Prunella Vulgaris (P. vulgaris) on the immune function in mice. The mice were randomly divided into one control group and three treatment groups of 10 mice each. The control group received pure water and the treatment groups received P. vulgaris extract at concentrations of 0.15, 0.30 and 0.90 g/kg BW orally for 30 days, respectively. Changes in cell immune function, non-specific immunity and humoral immunity function were evaluated. Active lymphocytes and T lymphocyte subsets were determined by fluorescence-activated cell sorting (FACS). Certain Serum concentrations of cytokines were measured by enzyme-linked immunosorbent assay (ELISA). The results showed that, for cell immune function, compared with the control group, foot pad thickness in high dose group increased significantly (*p*<0.01), whereas no significant difference in the proliferative ability of splenic lymphocytes was observed among all groups (*p*>0.05). For non-specific immunity, NK cell activity increased significantly in a dose-dependent manner in P. vulgaris treated mice (*p*<0.01), mononuclear-macrophage function in medium and high dose P. vulgaris treated mice were significantly higher than that of the control group (*p*<0.05). For humoral immunity, no significant differences were observed in terms of the half value of hemolysis (HC50), number of hemolytic plaques and serum IgG level (*p*>0.05). The percentage of active T and Th lymphocytes of mice peripheral blood in high dose group were significantly higher than that of the control group (*p*<0.01). There was no significant difference in serum levels of IL-1β, IL-4, IL-10 and IFN-γ among all of the four groups (*p*>0.05). The data indicated that 0.90 g/kg BW P. vulgaris extract (equivalent to 7.5 g/kg BW crude drug) had some effect on cellular immune function and non-specific immune function in mice.

## Introduction

P. vulgaris L. (Labiatae) belongs to Lamiaceae family Prunella genus. It is widely distributed in the temperate zone. Its Chinese name, Xia Ku Cao in pingyin, originates from a description that the herb dried and withered after the summer solstice. P. vulgaris has been used as a traditional Chinese medicine for a long time. According to the Chinese Pharmacopoeia [1], the dried fruit spike of P. vulgaris has effects of sedation, antifebrile and detumescence and is widely used for the treatment of thyroid gland malfunction, mastitis, and pulmonary tuberculosis in clinical practice [2], [3]. Modern pharmacological and clinical studies have suggested that P. vulgaris has anti-inflammatory, anti-bacterial, anti-viral and anti-tumor activities and these activities may be attributed to its immunomodulatory effect [4]–[6]. In Southeast Asia including the south of China, P. vulgaris is the main ingredient of herb tea. *In vivo* studies have suggested that herb tea consumption could alleviate the restraint stress-induced reduction of immune function in mice, manifested by alleviated lymphocyte impairment, increased amount and activity of lymphocytes such as natural killer (NK) cells and T lymphocytes [7]. *In vitro* studies revealed that both aqueous and ethanol extracts of P. vulgaris could promote lymphopoiesis and regulate the production of cytokines [8], [9]. However, most of the studies regarding the immunomodulatory effect of P. vulgaris were done *in vitro*, few have been reported *in vivo*. Therefore, in the current study, we investigated the effect of P. vulgaris on cellular immunity, humoral immunity and non-specific immunity in mice. By measuring the activities of lymphocytes and T lymphocytes subgroups as well as the serum level of humoral immunity-related cytokines such as IFN-γ, IL-1β, IL-10 and IL-4 after P. vulgaris administration, the result may provide some basis for potential mechanisms responsible for immunomodulatory effect of P. vulgaris.

## Materials and Methods

### Ethics Statement

This study was carried out in strict accordance with the recommendations in the Guide for the Care and Use of Laboratory Animals of the National Institutes of Health. The protocol was approved by the Committee on the Ethics of Animal Experiments of the Ethics Committee of Guangdong Provincial Centre for Disease Control and Prevention. All surgery was performed under sodium pentobarbital anesthesia, and all efforts were made to minimize suffering.

### Extraction

The extract of P. vulgaris was provided by Guangzhou Wanlaoji Pharmaceutical Co., Ltd. It was prepared from crude drug through water extraction followed by alcohol precipitation. One hundred g of crude drug can typically generate 11.76 g of extract; in other words, 1 g of extract is equivalent to 8.5 g of crude drug. The extract was maintained at 4°C. According to the Chinese Pharmacopoeia [1], the recommended daily consumption of P. vulgaris crude drug is 9–15 g. Assuming the body weight (BW) is 60 kg and using a recommended dosage of 15 g for calculation, the recommended daily consumption of P. vulgaris crude drug and extract are 0.25 and 0.03 g/kg BW, respectively.

### Animals

Female BALB/c mice (6–8 weeks, 16–19 g, SPF grade) [10] were selected from Guangdong Medical Laboratory animal center. The mice were quarantined for three days after arrival. Mice were housed in a temperature ranging from 20 to 25°C and humidity ranging from 40 to 70%. Feed and water were supplied ad libitum.

### Dose Planning and Test Substances Giving

The mice were randomly divided into one control group and three treatment groups (10 mice in each group). The control group received pure water and the treatment groups received P. vulgaris extract at 0.15, 0.30 and 0.90 g/kg BW, which were 5, 10 and 30 times of recommended dosage and equivalent to 1.25, 2.50 and 7.50 g/kg BW of crude drug respectively. To prepare the test substances for low, medium and high dose groups, 0.15,0.30 and 0.90 g P. vulgaris extract were dissolved in water to a final volume of 20.0 ml, respectively. The test substances were administrated intragastrically (0.2 ml/10 g BW) everyday for 30 days. The body weight was measured weekly. Twenty-four hours after the last administration, the mice were weighed and sacrificed.

### Chemicals and Instruments

#### Chemicals

Fluorescent beads (FITC-labeled), Concanavalin A (ConA), 3-(4,5-Dimethylthiazol-2-yl)-2,5-diphenyltetrazolium bromide (MTT), agar and bovine serum albumin (BSA) were purchased from Sigma (St. Louis, MO). Antibodies against mouse CD3 FITC, CD4 PE, CD8 APC, CD19 PE, CD25 PE-Cy5 and CD25 APC were from eBioscience (San Diego, CA). Heamolysin was from Becton Dickinson (San Jose, CA). ELISA kits detecting Mouse IgG, IL-1β, IL-4, IFN-γ and IL-10 were purchased from R&D (Minneapolis, MN).

#### Instruments

VL-5BS biological safety cabinet was from Tsaohsin (Taibei, Taiwan). FACS Calibur flow cytometer was from Becton Dickinson (San Jose, CA, USA). Varioskan Flash Spectral Scanning Multimode Reader was from Thermo Scientific (Vantaa, FI). AutoFlow CO_2_ incubator was from NuAire (Plymouth, MN, USA). Digital sonofier 450 was from Branson (MX). CELL-DYN 3700 hematology analyzer was from Abbott (Abbott Park, IL, USA). CASY DT cell counter was from Schärfe System (DE).

### Measurement of Spleen and Thymus Indexes

After the last measurement of body weight, mice were sacrificed by cervical dislocation. The spleens and thymus organs were removed immediately and the wet weight was measured. The spleen and thymus indexes were calculated as the wet weight of spleen and thymus divided by the body weight, respectively.

### ConA-induced Mouse Splenic Lymphocyte Proliferation Assay [10]

To prepare splenocyte suspensions, spleens were removed in a sterile environment and put into a petri dish with sterile Hank’s buffered salt solution (HBSS). Each spleen was teased apart by sterile forceps and pushed through 200 BSS mesh. The resultant single splenocytes were washed with HBSS and centrifuged (1000 r/min, 10 min) three times. The cells were then re-suspended in 2 mL RPMI 1640 complete media. The live cells were counted by trypan blue exclusion assay (the percentage of which should be above 95%) and the cell number was adjusted to 3×10^6^ cells/mL. One mL splenocyte suspension was incubated with or without 75 µL ConA solution (equivalent to 7.5 µg/mL) in a 24-well plate at 37°C with 5% CO_2_ for 72 hours. After 68 hours, 0.7 mL media was removed and replaced with 0.7 mL serum-free RPMI 1640 media plus 50 µL 5 mg/mL MTT. Four hours later (t = 72 h), 1 mL acidic isopropanol was added to dissolve the purple formazan product. The final solution was added into a 96-well plate in triplicate. The optical density (OD) value was measured by a microplate reader set to 570 nm.

### Delayed Type Hypersensitivity (DTH) Assay [10]

Twenty-six days into the experiment, mice were immunized by 0.2 mL 2% sheep red blood cells (SRBC) (v/v) via intraperitoneal injection. Four days later (t = 30 d), the thickness of the left hind foot pad was measured by cornier caliper. Each sample was measured three times at the same place and the mean was taken. The spot of measurement then received 20 µL 20% SRBC (v/v) via subcutaneous injection. An additional 24 hours later, the thickness of left hind foot pad was again measured. The difference between two measurements was used as a measure of DTH. The result is considered positive if the value of experimental group is significantly higher than that of control group.

### Measurement of Serum Hemolysin Level [10]

Twenty-six days into the experiment, mice were immunized by SRBC. On the last day (t = 30 d), blood was collected from the angular vein before mice were sacrificed. Serum was separated and diluted with salicylic acid (SA) buffer at a 1∶100 ratio. 100 µL diluted serum was added into a 96-well plate, followed by 50 µL 10% SRBC and 100 µL complement (diluted with SA buffer at 1∶8 ratio). For the blank control, SA buffer was used instead of diluted serum. The plate was incubated in a 37°C water bath for 30 min. After incubation, the plate was centrifuged at 2000 r/min for 10 min. Fifty µL supernatant was transferred into a new 96-well plate, followed by addition of 150 µL buffer A (1 g/L NaHCO3, 0.2 g/L KFeCN, 0.05 g/l KCN). 12.5 µL 10% SRBC was then added, again followed by buffer A to a final volume of 200 µL. The plate was incubated at room temperature for 10 min. The OD value was measured by a microplate reader set to 540 nm. The half value of hemolysis (HC_50_) was calculated as follows:
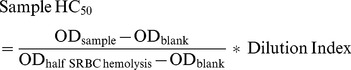
(1)


### Measurement of Antibody Production by Splenocytes [10]

Twenty-six days into the experiment, mice were immunized by SRBC. On the last day (t = 30 d), mice were sacrificed by cervical dislocation. Splenocyte suspensions (3×10^6^ cells/mL) were prepared as described above. Fifty µL 20% SRBC (v/v in saline) and 200 µL splenocyte suspension were mixed with 0.5 mL agar (5 g/mL in HBSS, pH 7.2–7.4), and added into a 6-well plate coated with 1 mL/well agar (5 g/L in sterile saline). Each sample was tested in duplicate. The plate was incubated at 37°C with 5% CO_2_ for 1 hour. 500 µL diluted complement (v/v, 1∶10 in complete media) was then added into each well, followed by further incubation for another 2 hours. After incubation, hemolytic plaques were counted by automatic image analyzer. The number of hemolytic plaques in each sample is presented as mean of duplicate/10^6^ splenocytes.

### Measurement of Serum Immunoglobulin G (IgG) Level by Enzyme-linked Immunosorbent Assay (ELISA)

Mice were anesthetized and blood was collected via the abdominal vein. Serum was separated by centrifugation and used for measurement of IgG concentration by ELISA according to the product manual. Briefly, 50 µL standard solution or sample was added into a 96-well plate (provided in the kit), followed by 25 µL enzyme-linked avidin. The plate was covered with adhesive strip and incubated at 37°C for 1 hour. The plate was then washed with pre-diluted washing buffer 5 times and 50 µL each of substrate I and substrate II were added into each well. The plate was incubated at room temperature and protected from light for 15 min. After incubation, 50 µL stop solution was added. The OD value was measured by a microplate reader set to 450 nm within 15 min. The IgG concentration in each sample was calculated according to the standard curve generated by standard solution. The result was considered positive if the value of the experimental group was significantly higher than that of the control group.

### Measurement of NK Cell Activity by Lactate Dehydrogenase (LDH) Releasing Assay [10]

Routinely subcultured target cells were washed three times with HBSS. Sixty µL target cells were diluted with 6 mL cell counting buffer for cell number counting by automatic cell counter. The target cell number was then adjusted to 4×10^5^ cells/mL in RPMI 1640 complete media. Splenocyte suspensions were prepared as described above. Six µL splenocyte suspension was mixed with 6 mL cell counting buffer for cell number counting by automatic cell counter. The number of splenocytes was then adjusted to 2×10^7^ cells/mL in RPMI 1640 complete media. 100 µL target cells and 100 µL splenocytes (effector cell) (ratio of effector cells to target cell is 50∶1) were added into a 96-well plate. 100 µL target cell plus 100 µL media served as target cell spontaneous release control, whereas 100 µL target cell plus 100 µL 1% NP40 served as target cell maximum release control. The plates were incubated at 37°C in 5% CO_2_ for 4 h. After incubation, the plate was centrifuged at 1500 r/min for 5 min. 100 µL supernatant was transferred into a new 96-well plate, followed by addition of 100 µL LDH substrate. After 3 min, 30 µL l mol/L HCI was added to stop the reaction. The OD values were measured by a microplate reader set to 490 nm. NK cell activity was calculated and transformed as Formula (2). The result was considered positive if the transformed value of the experimental group was significantly higher than the transformed value of the control group.
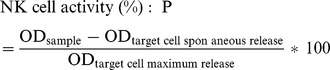


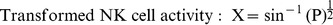
(2)


### Detection of Phagocytic Activity of Peritoneal Macrophages by Flow Cytometry

Twenty-six days into the experiment, mice were immunized by SRBC. On the last day (t = 30 d), the mice were sacrificed by cervical dislocation. To collect peritoneal macrophages, 3 mL HBSS (containing 5% fetal bovine serum) was directly injected into the peritoneal cavity. After peritoneal massage (40 times), the peritoneal cavity was surgically opened and 2 mL peritoneal cavity fluid was collected. The peritoneal macrophages were then collected by filtration onto a 70 um filter. 100 µL FITC-labeled fluorescent beads was added into 10 mL PBS containing 1% BSA, incubated in a 37°C water bath for 30 min, followed by sonication for 5 min. Pretreated fluorescent beads were then mixed with peritoneal macrophages (200 µL fluorescent beads for every mL macrophages at a concentration of 4∼6×10^5^ cells/mL) and added into a 6-well plate. The plate was incubated at 37°C for 1 hour. After incubation, the supernatant was removed and the wells were gently washed with PBS. The adherent cells were then collected and filtered onto a 70 µm filter, followed by FACS analysis. The phagocytic activity of peritoneal macrophages was presented as percentage of phagocytosis (which needs data transformation) and phagocytic index calculated as Formula (3) and (4).



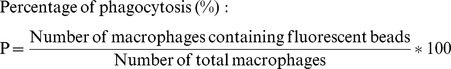


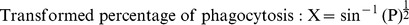
(3)

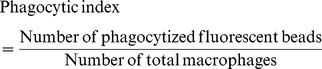
(4)


### Analysis of Active Lymphocytes and T Lymphocyte Subsets

Activated lymphocytes and lymphocyte subgroups in peripheral blood were analyzed by fluorescence-activated cell sorting (FACS) [11]. CD3 was used as a marker for mature T lymphocytes; CD4 and CD8 were used as markers for helper T lymphocytes (Th) and cytotoxic T lymphocytes (Tc or CTL), respectively; CD19 was used as a marker for B lymphocytes; and CD25 was used as a marker for activated lymphocytes. Before mice were sacrificed, blood was collected from the angular vein and prevented from coagulation by heparin. 100 µL anticoagulated blood was mixed with fluorescence-labeled mouse monoclonal antibodies against the above-mentioned markers and incubated at room temperature and protected from light for 20 min. After incubation, 2 mL FACS red blood cell lysis buffer was added into each tube and incubated at room temperature and protected from light for another 20 min. After washing with phosphate buffered saline, the samples were analyzed by FACS (Figure S1 and Figure S2).

### Measurement of Concentrations of Serum Cytokines IL-1β, IFN-γ, IL-4 and IL-10 by ELISA

Mice were anesthetized and blood was collected via the abdominal vein. Serum was separated by centrifugation and used for the measurement of cytokine concentrations by ELISA according to the product manual. For measurement of IL-4 and IL-10, 100 µL standard solution or serum sample were added to the ELISA plate. For measurement of IFN-γ and IL-1β, 50 µL standard solution or serum sample were added. Then, 50 µL enzyme-linked avidin was added into each well. The plate was covered with adhesive strip and incubated at 37°C for 1 hour. The plate was then washed five times with pre-diluted washing buffer, followed by addition of 50 µL each of substrate I and substrate II into each well and incubation at room temperature with protection from light for 15 min. After incubation, 50 µL stop solution was added into each well. The OD values were measured by a microplate reader set to 450 nm within 15 min. The concentration of each cytokine was calculated according to the standard curve generated by the corresponding standard solution.

### Statistical Analysis

Values were expressed as mean ± SD (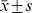). Statistical analysis were performed using one-way ANOVA. Then the least significant difference (LSD) test was used to compare each dose group with the control group. Differences with a *p*<0.05 were considered statistically significant.

## Results

### Effect of P. vulgaris on Mice Spleen and Thymus

**Figure 1:**
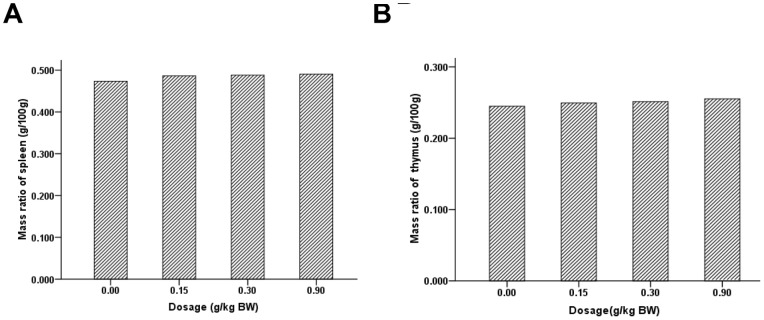
The effect of P. vulgaris on mice spleen and thymus indexes. The spleen and thymus indexes were calculated as the wet weight of spleen and thymus divided by the body weight respectively for P. vulgaris exact treated (0.00, 0.15, 0.30, 0.90 g/kg BW) mice for 30 days. The data were expressed as mean±SD of each group (n = 10 per group). There was no significant difference in spleen index (Fig. 1A) and thymus index (Fig. 1B) among four groups (*p*>0.05).

No pathological changes such as swelling, atrophy or bleeding were observed by visual observation in P. vulgaris exact (0.00, 0.15, 0.30, 0.90 g/kg BW) treated mice for 30 days. The spleen and thymus indexes were calculated as the wet weight of spleen and thymus divided by the body weight, respectively. As the result of statistical analysis, the data were expressed as mean±SD of each group (n = 10 per group). There was no significant difference in spleen index (Fig. 1A) and thymus index (Fig. 1B) among four groups (p>0.05). The results of the spleen and thymus indexes suggest that P. vulgaris has no significant effect on the major immune organs in mice.

### Effect of P. vulgaris on Mice Cellular Immune Function

To explore the the effect of P. vulgaris on mice cellular immune function, Delayed type hypersensitivity (DTH) and ConA-induced splenic lymphocyte proliferation were tested in mice treated with P. Vulgaris exact (0.00, 0.15, 0.30, 0.90 g/kg BW) for 30 days. The data were expressed as mean±SD of each group (n = 10 per group). The increase in foot pad thickness of mice immunized with SRBC was examined to represent the DTH. As the result, the thickness of the mice foot pad increased in a dose dependent manner. The increase in foot pad thickness increased significantly (*p*<0.01) among these four groups. Further analysis revealed that a statistically significant difference was observed between the high dose group (0.75±0.11) and the control group (0.60±0.07) (*p*<0.01). While there were no significant differences between low or medium dose P. vulgaris exact treated mice with the control group (*p*>0.05). ConA-induced splenic lymphocyte proliferation was tested by MTT assay. There was no obvious difference in the splenic lymphocyte proliferation ability among the four groups (*p*>0.05) (Table 1). The results suggest that high concentrations of P. vulgaris may have the potential effect on mice cellular immune function.

**Table 1:** The effect of P vulgaris on mice cellular immune function (mean±SD, n = 10).

Group	Dosage (g/kg BW)	Increase in foot pad thickness (mm)	Spleniclymphocyteproliferation
Control group	0.00	0.60±0.07	0.37±0.11
Low-dosage group	0.15	0.63±0.09	0.34±0.12
Medium-dosage group	0.30	0.68±0.14	0.42±0.06
High-dosage group	0.90	0.75±0.11**	0.39±0.09

Note: Compared to the control group,

** indicates P<0.01.

The tested doses are of the extract of P. vulgaris.

### Effect of P. vulgaris on Mice Non-specific Immunity

#### Mononuclear-macrophage function

The mononuclear-macrophage function was evaluated by detecting the phagocytic activity of peritoneal macrophages by flow cytometry in P. vulgaris exact treated mice with different dosage (0.00, 0.15, 0.30, 0.90 g/kg BW) for 30 days. The percentage of phagocytosis (which needs data transformation) and phagocytic index of mononuclear-macrophage of each mice was calculated as Formula (3) and (4) according to the number of macrophages containing fluorescent beads analyzed by FACS. The data were expressed as mean±SD of each group (n = 10 per group).Both the transformed percentage of phagocytosis and the phagocytic index increased in a dose-dependent manner in mice treated with P. vulgaris. The phagocytic index of the medium (1.32±0.19) and high (1.33±0.22) dose group were significantly higher than that of the control group (1.05±0.12) (p<0.05). The transformed percentage of phagocytosis of the medium (0.89±0.06) and high (0.89±0.09) dose group remained significantly higher than that of the control group (0.80±0.05) (*p*<0.05). While there was no obvious difference in the transformed percentage of phagocytosis and the phagocytic index between the low dose group and the control group (*p*>0.05) (Table 2). The results suggest that medium and high dosage of P. vulgaris may have some effect on mice Mononuclear-macrophage function.

**Table 2:** The effect of P vulgaris on mice mononuclear-macrophage function (mean±SD, n = 10).

Group	Dosage (g/kg BW)	Phagocytic index	Transformed percentage of phagocytosis
Control group	0.00	1.05±0.12	0.80±0.05
Low-dosage group	0.15	1.13±0.11	0.83±0.05
Medium-dosage group	0.30	1.32±0.19*	0.89±0.06*
High-dosage group	0.90	1.33±0.22*	0.89±0.09*

Note: Compared to the control group,

* indicates P<0.05.

#### NK cell activity

The mice were treated with increasing concentrations (0.00, 0.15, 0.30, 0.90 g/kg BW) of P. vulgaris exact for 30 days. Then the NK cell activity was evaluated by the LDH releasing assay. Transformed NK cell activity of each sample was calculated and transformed as the Formula (2) according to the OD values measured by microplate reader. The data were expressed as mean±SD of each group (n = 10 per group).Transformed NK cell activity increased in a dose-dependent manner in P. vulgaris treated mice. Statistically significant differences were observed between all three experimental groups and the control group (*p*<0.01). As shown in Table 3,the transformed NK cell activity of the medium (0.57±0.06) and high (0.62±0.07) dose group were significantly higher than that of the control group (0.49±0.06)(*p*<0.01). The transformed NK cell activity of low dose group (0.54±0.03 ) remained significantly higher than that of the control group (*p*<0.05). The results suggest that low, medium and high dosage of P. vulgaris may have some effect on mice NK cell activity.

**Table 3:** The effect of P vulgaris on mice NK cell activity (mean±SD, n = 10).

Group	Dosage (g/kg BW)	NK cell activity (%)	Transformed NK cell activity
Control group	0.00	22.07±4.73	0.49±0.06
Low-dosage group	0.15	26.36±3.05	0.54±0.03*
Medium-dosage group	0.30	29.27±4.99**	0.57±0.06**
High-dosage group	0.90	33.59±6.28**	0.62±0.07**

Note: Compared to the control group,

* vindicates P<0.05,

** indicates P<0.01.

### Effect of P vulgaris on Mice Humoral Immunity Function

To explore the the effect of P. vulgaris on mice humoral immunity function, the mice serum hemolysin level (HC_50_), hemolytic plaques (duplicate/10^6^ splenocytes) indicating antibody production of splenocytes, as well as serum IgG level were evaluated in mice treated with increasing concentrations (0.00, 0.15, 0.30, 0.90 g/kg BW) of P. Vulgaris exact for 30 days. The data were expressed as mean±SD of each group (n = 10 per group).No significant difference was detected among the three experimental groups and a control group in terms of HC_50_, the number of hemolytic plaques and serum IgG level (*p*>0.05) (Table 4).

**Table 4:** The effect of P vulgaris on mice humoral immunity (mean±SD, n = 10).

Group	Dosage (g/kg BW)	HC_50_	hemolytic plaque (duplicate/10^6^ splenocytes)	Serum IgG level (µg/ml)
Control group	0.00	12.19±2.77	16.03±2.65	37.30±3.92
Low-dosage group	0.15	12.89±1.77	16.25±3.21	41.35±7.47
Medium-dosage group	0.30	13.45±2.77	16.09±3.25	40.20±5.39
High-dosage group	0.90	13.08±1.77	18.08±3.34	39. 55±4.94

### Effect of P. vulgaris on the Lymphocytes and T Lymphocyte Subsets Activity in Mice Peripheral Blood

The mice received increasing concentration (0.0, 0.15, 0.30, 0.90 g/kg BW) of P. Vulgaris exact for 30 days Then the active lymphocytes and the T lymphocyte subsets in mice peripheral blood were analyzed by FACS. According to the ratio of the active T, Th and Tc lymphocytes of each sample obtained by the flow cytometry, the detection result for each dose group can then be compared by statistical analysis. The data were expressed as mean±SD of each group (n = 10 per group).Compared with the control group, the percentages of both active T lymphocytes (CD3^+^CD25^+^) and Th cells (CD3^+^CD4^+^CD8^−^CD25^+^) increased in a dose-dependent manner in P. vulgaris treated mice. In terms of the percentages of active T lymphocytes, the high dose group (CD3^+^CD25^+^ = 8.15±0.95% ), but not the low or medium dose group, is significantly higher than the control group (CD3^+^CD25^+^ = 6.38±0.66% )(*p*<0.01). The active Th lymphocytes in high dose group (CD3^+^ CD4^+^CD8^−^ CD25^+^ = 6.53±0.91% ) remained significantly higher than that of the control group (CD3^+^ CD4^+^CD8^−^ CD25^+^ = 3.78±0.57% ) (*p*<0.01). While there was no significant difference in the percentage of active B lymphocytes (CD19^+^CD25^+^) and Tc lymphocytes (CD3^+^CD4^−^CD8^+^CD25^+^) among the four groups (*p*>0.05).(Table 5) The results suggest that the high dose P. vulgaris may have some effect on the T and Th lymphocytes activity in mice peripheral blood.

**Table 5:** The effect of P vulgaris on mice active lymphocytes and T lymphocyte subsets in mice peripheral blood (mean±SD, n = 10).

Group	Dosage (g/kg BW)	CD3^+^ CD25^+^ (%)	CD3^+^CD4^+^CD8^−^ CD25^+^(%)	CD3^+^CD4^−^CD8^+^ CD25(%)	CD19^+^ CD25^+^ (%)
Control group	0.00	6.38±0.66	3.78±0.57	0.15±0.02	0.28±0.05
Low-dosage group	0.15	6.70±0.87	4.27±0.86	0.17±0.04	0.28±0.13
Medium-dosage group	0.30	6.73±0.82	4.95±1.53	0.16±0.07	0.32±0.12
High-dosage group	0.90	8.15±0.95 **	6.53±0.91**	0.20±0.07	0.26±0.10

Note: Compared to the control group,

** indicates P<0.01.

### Effect of P vulgaris on Serum Concentrations of Cytokines

**Figure 2:**
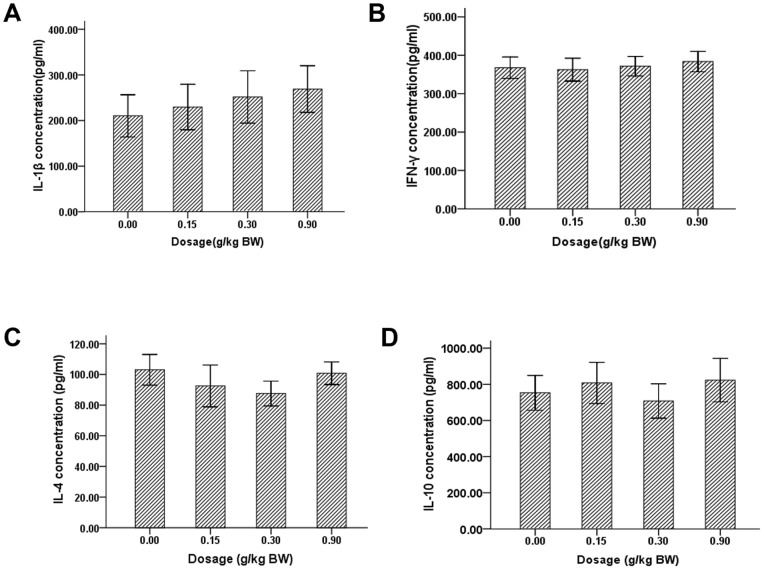
The effect of P vulgaris on mice serum concentrations of cytokines. The serum levels of IL-1β, IFN-γ, IL-4 and IL-10 in P. vulgaris exact treated (0.00, 0.15, 0.30, 0.90 g/kg BW) mice for 30 days were detected by ELISA according to the manual of ELISA kits. The data were expressed as mean±SD of each group (n = 10 per group). No significant difference in serum levels of IL-1β (Fig. 2.A), IFN-γ (Fig. 2B), IL-4(Fig. 2C) and IL-10 (Fig. 2D) was observed among these four groups of mice (*p*>0.05).

Serum concentrations of different cytokines including IL-1β, IFN-γ, IL-4 and IL-10 in P. vulgaris exact (0.00, 0.15, 0.30, 0.90 g/kg BW) treated mice were tested by ELISA according to the manual of ELISA kits. The data were expressed as mean±SD of each group (n = 10 per group).The serum concentration of IL-1β tended to increase in a dose-dependent manner in P. vulgaris treated mice. However, no significant difference in serum levels of IL-1β (Fig. 2A), IFN-γ (Fig. 2B), IL-4 (Fig. 2C) and IL-10 (Fig. 2D) was observed among four groups (*p*>0.05).

## Discussion

Studies have suggested that the anti-inflammatory and antitumor activities of P. vulgaris are closely related to its immunomodulatory effect [4], [12], [13]. Our current study showed that 0.90 g/kg BW P. vulgaris extract had some effect on mice cellular immune function and non-specific immune function. While 0.30 and 0.15 g/kg BW Prunella extract may have some potential effect on mice cellular immune function and non-specific immune function. For non-specific immunity, both NK cell activity and macrophage function increased significantly in a dose-dependent manner in P. vulgaris treated mice. Compared with the control group, significant increases in NK cell activity were observed in all three experimental groups and significant increases in macrophage function were observed in medium and high dose groups. These results suggest that P. vulgaris is able to effect mice non-specific immunity in a dose-dependent manner, which is consistent with other studies. For example, aqueous extract of P. vulgaris enhanced macrophage function by regulating the NF-κB pathway in RAW 264.7 mouse macrophage cell line [14], [15]. Compound Ajuga decumbens *Thunb*, another species in the same family as P. vulgaris, showed similar ability to enhance phagocytic activity of mouse macrophages in a dose-dependent manner [16]. Moreover, studies have indicated that the bioactive components of P. vulgaris such as polysaccharides, oleanolic acid and ursolic acid may be responsible for boosting non-specific immunity in mice, presented as a dose-dependent increase of phagocytic activity of mononuclear-macrophages and IL-1β secretion from macrophages [17]–[19]. Our current study also suggests that P. vulgaris may affect mice cell immune function, manifested by a dose-dependent increase in foot pad thickness in mice treated with P. vulgaris. There was a significant difference between high dose group and the control group in terms of foot pad thickness. It has been reported that the aqueous extract of P. vulgaris can stimulate mouse T lymphocyte proliferation [8]. Polysaccharides from P. vulgaris were shown to affect cell immune function in mice and induce the secretion of interferon (IFN) in a dose-dependent manner [20]. Furthermore, no influence of P. vulgaris on mouse humoral immunity was observed in the current study, evident by no significant difference between the experimental groups and the control group in terms of serum hemolysin levels, antibody producing splenic lymphocytes and serum IgG levels.

The activity of T lymphocyte subsets is an important indicator of immune homeostasis [21]. It was reported that intragastric administration of P. vulgaris capsules at a concentration of 0.21–0.86 g/kg·d for 30 days induced up-regulation of certain T lymphocyte subgroups in rats [3]. The current study showed that P. vulgaris treatment enhanced the activity of T lymphocytes in general and its subgroup Th cells; in contrast, it had minimal effects on B lymphocytes and Tc cells, another subset of T lymphocytes. The thickness of the mice foot pad and the percentages of active T and Th lymphocytes increased in a dose-dependent manner in P. vulgaris treated mice. Compared with the control group, the high dose group showed significantly higher percentage of active T lymphocytes and its subgroup Th cells, further proving that P. vulgaris is able to effect the cellular immune function in mice. No significant changes in B lymphocytes and Tc cells were observed among the four groups, which is consistent with the finding that P. vulgaris had minimal effect on mouse humoral immunity.

## Conclusion

In conclusion, 0.90 g/kg BW P. vulgaris extract (equivalent to 7.50 g/kg BW crude drug) had some effect on cellular immune function and non-specific immune function in mice.

## Supporting Information

Figure S1 **Flow cytometry detection of the active T lymphocyte subsets.** These pictures are the detection results of a sample. I gate in Fig. S1A indicates the lymphocyte region. The labeled cells were acquired in the two-dimensional Dot-Plot graph by Forward scatter (FSC) and Side scatter (SSC). In Fig. S1 B–D, the right upper quadrant and lower right quadrant were lymphocyte region. Right upper quadrant represents active lymphocyte region, while lower right quadrant represents non active lymphocyte region. Active T, Th, TC lymphocytes were detected through three fluorescent antibodies of FITC-CD3/PE-CD19/APC-CD25. The right upper quadrant in Fig. S1B, Fig. S1C and Fig. S1D was respectively the region of active T(CD3^+^ CD25^+^), Th(CD3^+^ CD4^+^CD8^−^ CD25^+^) and Tc(CD3^+^ CD4^−^CD8^+^ CD25^+^) lymphocytes. According to the ratio of the active T, Th and Tc lymphocytes of each sample obtained by flow cytometry, the detection result for each dose group can then be analyzed.
(TIF)

Figure S2 **Flow cytometry detection of the active B lymphocyte.** These pictures are the detection results of a sample. I gate in Fig. S2A indicates the lymphocyte region. The labeled cells were acquired in the two-dimensional Dot-Plot graph, the right upper quadrant and lower right quadrant in Fig. S2 B was lymphocyte region. Active B lymphocytes were detected through two fluorescent antibody simultaneously of PE-CD19/APC-CD25. The right upper quadrant in Fig. S2B was the region of active B lymphocytes (CD19^+^ CD25^+^). According to the ratio of the active B lymphocytes of each sample obtained by flow cytometry, the detection result for each dose group can then be analyzed.
(TIF)
