# Supplementary figures and images for: Effects of Prunella vulgaris on the Mice Immune Function
Source: PLoS One. 2013 Oct 30;8(10):e77355. doi: 10.1371/journal.pone.0077355 (PMC3813705; doi:10.1371/journal.pone.0077355)

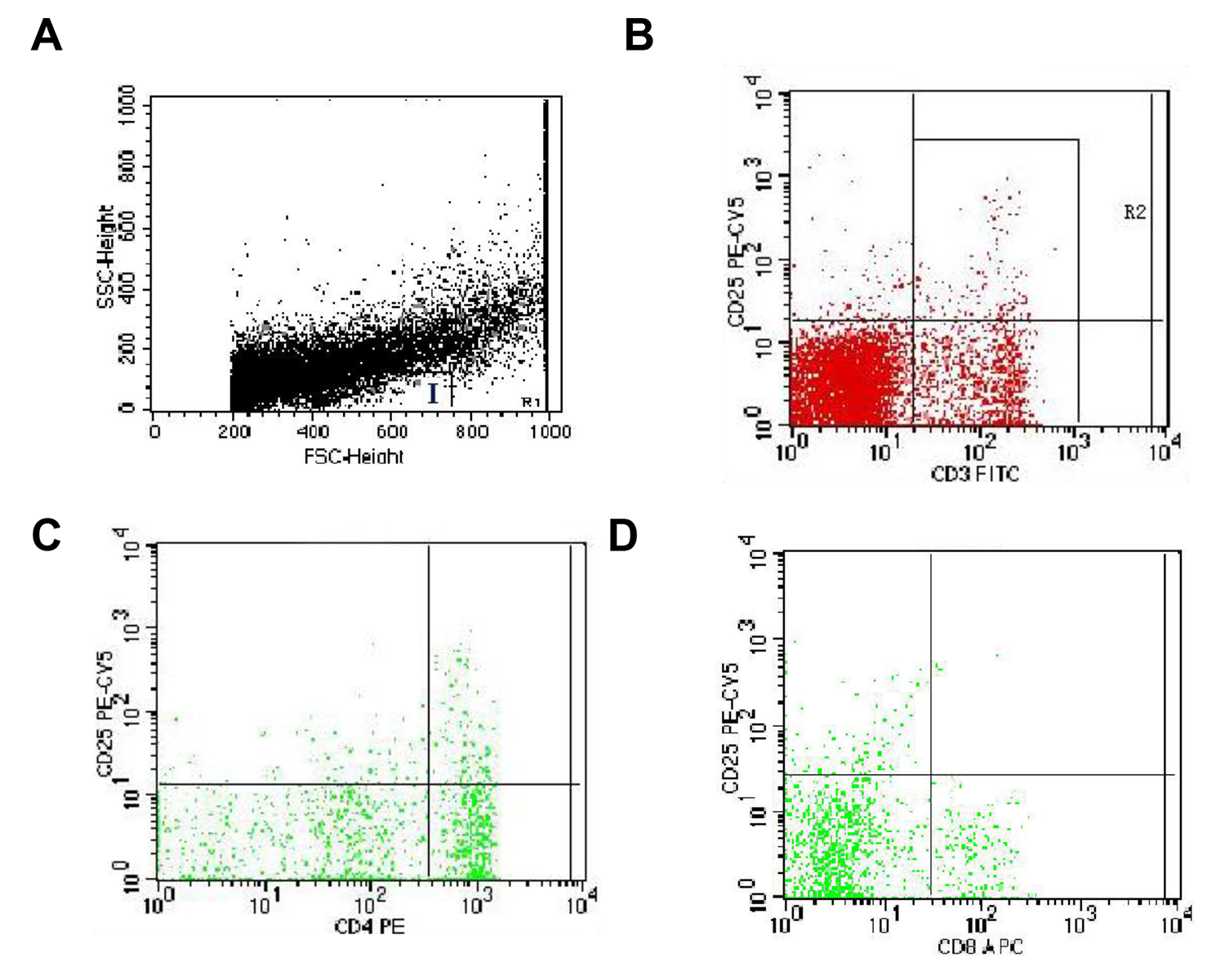

Supplement: Figure S1 — Flow cytometry detection of the active T lymphocyte subsets. These pictures are the detection results of a sample. I gate in Fig. S1A indicates the lymphocyte region. The labeled cells were acquired in the two-dimensional Dot-Plot graph by Forward scatter (FSC) and Side scatter (SSC). In Fig. S1 B–D, the right upper quadrant and lower right quadrant were lymphocyte region. Right upper quadrant represents active lymphocyte region, while lower right quadrant represents non active lymphocyte region. Active T, Th, TC lymphocytes were detected through three fluorescent antibodies of FITC-CD3/PE-CD19/APC-CD25. The right upper quadrant in Fig. S1B, Fig. S1C and Fig. S1D was respectively the region of active T(CD3+ CD25+), Th(CD3+ CD4+CD8− CD25+) and Tc(CD3+ CD4−CD8+ CD25+) lymphocytes. According to the ratio of the active T, Th and Tc lymphocytes of each sample obtained by flow cytometry, the detection result for each dose group can then be analyzed. (TIF) [file pone.0077355.s001.tif]

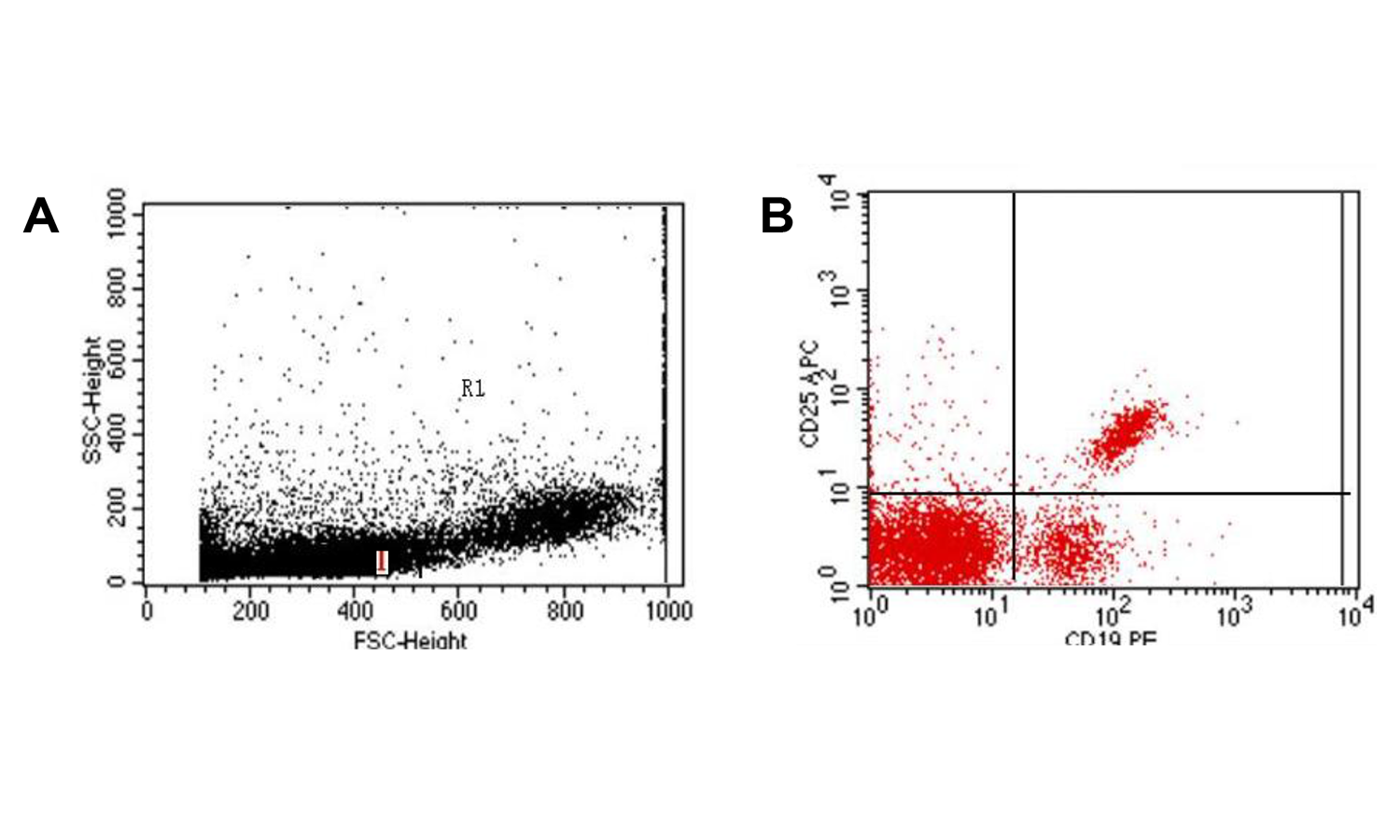

Supplement: Figure S2 — Flow cytometry detection of the active B lymphocyte. These pictures are the detection results of a sample. I gate in Fig. S2A indicates the lymphocyte region. The labeled cells were acquired in the two-dimensional Dot-Plot graph, the right upper quadrant and lower right quadrant in Fig. S2 B was lymphocyte region. Active B lymphocytes were detected through two fluorescent antibody simultaneously of PE-CD19/APC-CD25. The right upper quadrant in Fig. S2B was the region of active B lymphocytes (CD19+ CD25+). According to the ratio of the active B lymphocytes of each sample obtained by flow cytometry, the detection result for each dose group can then be analyzed. (TIF) [file pone.0077355.s002.tif]
